# Microbial Sulfate Reduction Potential in Coal-Bearing Sediments Down to ~2.5 km below the Seafloor off Shimokita Peninsula, Japan

**DOI:** 10.3389/fmicb.2016.01576

**Published:** 2016-10-05

**Authors:** Clemens Glombitza, Rishi R. Adhikari, Natascha Riedinger, William P. Gilhooly, Kai-Uwe Hinrichs, Fumio Inagaki

**Affiliations:** ^1^Department of Biosciences, Center for Geomicrobiology, Aarhus UniversityAarhus, Denmark; ^2^MARUM Center for Marine Environmental Sciences, University of BremenBremen, Germany; ^3^Boone Pickens School of Geology, Oklahoma State UniversityStillwater, OK, USA; ^4^Department of Earth Sciences, Indiana University-Purdue University IndianapolisIndianapolis, IN, USA; ^5^Kochi Institute for Core Sample Research, Japan Agency for Marine-Earth Science and TechnologyKochi, Japan; ^6^Research and Development Center for Ocean Drilling Science, Japan Agency for Marine-Earth Science and TechnologyYokohama, Japan; ^7^Research and Development Center for Submarine Resources, Japan Agency for Marine-Earth Science and TechnologyYokosuka, Japan

**Keywords:** sulfate reduction, deep biosphere, coal, lignite, IODP Expedition 337, hydrogenase, pyrite, isotopes

## Abstract

Sulfate reduction is the predominant anaerobic microbial process of organic matter mineralization in marine sediments, with recent studies revealing that sulfate reduction not only occurs in sulfate-rich sediments, but even extends to deeper, methanogenic sediments at very low background concentrations of sulfate. Using samples retrieved off the Shimokita Peninsula, Japan, during the Integrated Ocean Drilling Program (IODP) Expedition 337, we measured potential sulfate reduction rates by slurry incubations with ^35^S-labeled sulfate in deep methanogenic sediments between 1276.75 and 2456.75 meters below the seafloor. Potential sulfate reduction rates were generally extremely low (mostly below 0.1 pmol cm^−3^ d^−1^) but showed elevated values (up to 1.8 pmol cm^−3^ d^−1^) in a coal-bearing interval (Unit III). A measured increase in hydrogenase activity in the coal-bearing horizons coincided with this local increase in potential sulfate reduction rates. This paired enzymatic response suggests that hydrogen is a potentially important electron donor for sulfate reduction in the deep coalbed biosphere. By contrast, no stimulation of sulfate reduction rates was observed in treatments where methane was added as an electron donor. In the deep coalbeds, small amounts of sulfate might be provided by a cryptic sulfur cycle. The isotopically very heavy pyrites (δ^34^S = +43‰) found in this horizon is consistent with its formation via microbial sulfate reduction that has been continuously utilizing a small, increasingly ^34^S-enriched sulfate reservoir over geologic time scales. Although our results do not represent *in-situ* activity, and the sulfate reducers might only have persisted in a dormant, spore-like state, our findings show that organisms capable of sulfate reduction have survived in deep methanogenic sediments over more than 20 Ma. This highlights the ability of sulfate-reducers to persist over geological timespans even in sulfate-depleted environments. Our study moreover represents the deepest evidence of a potential for sulfate reduction in marine sediments to date.

## Introduction

Sulfate reduction is a globally important microbial process in anoxic marine sediments (Canfield, 1991; Jørgensen and Kasten, 2006; Bowles et al., 2014). It is an important pathway for carbon recycling in the seabed and represents the predominant terminal process of carbon remineralization in sulfur-rich marine shelf sediments (Jørgensen, 1982). From the overlaying seawater, sulfate diffuses downwards into the sediments where it can serve as an electron acceptor for microbial sulfate reduction. Diffusion and microbial turnover result in a concentration gradient from ~28 mmol L^−1^ at the sediment surface down to a few μmol L^−1^, which determines the bottom of the sulfate zone (Froelich et al., 1979; Berner, 1981; Jørgensen and Kasten, 2006). The sulfate methane transition zone (SMTZ) marks the end of the sulfate zone and the onset of the methane zone, where methane is diffusing upwards from deeper sediments in which methanogens predominate (Iversen and Jørgensen, 1985). At the SMTZ, methane is oxidized by methane-oxidizing sulfate-reducing microorganisms (Barnes and Goldberg, 1976; Treude et al., 2005; Caldwell et al., 2008). In the sulfate reduction zone, sulfate-reducing microorganisms typically outcompete methanogens for shared energy substrates, such as H_2_ and acetate, by bringing the concentrations of these compounds to such low levels that methanogenesis is not thermodynamically feasible (Hoehler et al., 1998, 2001). Nonetheless, small populations of methanogens are ubiquitous in sulfate-reducing sediment, and typically consist of methanogens that are capable of metabolizing “non-competitive substrates,” i.e., C_1_ compounds, such as methanol, methylamines, and methyl sulfides, which are not utilized by most sulfate reducers (Oremland and Polcin, 1982; Orsi et al., 2013; Watkins et al., 2014). Similarly, in recent studies, sulfate reduction was also detected in methane zones, operating at low background concentrations of sulfate (Leloup et al., 2006; Holmkvist et al., 2011; Treude et al., 2014; Brunner et al., 2016; Orsi et al., 2016). This shows that although there is a general zonation of predominant microbial processes in the sediment column determined by pore water chemistry and thermodynamics, this zonation is not absolute and exceptions are common.

When substrate concentrations and concomitantly the available energy for the microbial activity decrease, microbes slow down their metabolism, and biomass turnover to generation times of several 100 years (Lomstein et al., 2012; Hoehler and Jørgensen, 2013). However, slow turnover rates and long generation times also reduce the speed of necessary cellular maintenance processes, such as DNA and protein repair (Johnson et al., 2007; Morita et al., 2010; Lever et al., 2015). Increasing burial depth does not only lead to exhaustion of energy-rich substrates but also leads to increasing damage rates as sediment temperature increases (Lever et al., 2015). Recently it was discovered that, despite the slow metabolic rates in the deep biosphere, the expression of DNA repair genes increases with sediments depth (Orsi et al., 2013), highlighting the increased importance of damage repair for microorganisms in deeply buried sediments. Consequently, there is a balance of available substrates providing the metabolic energy for necessary maintenance of basic cell functions and environmentally induced damage that marks the boundary between life and death. A potential strategy for microbial life to cope with periods of starvation is the formation of endospores (Schrenk et al., 2010; Lomstein et al., 2012). In this dormant stage of life, the cell has formed a metabolically inactive endospore that will only germinate when conditions become more supportive of growth. However, it is questionable if such a strategy helps to increase survival as damage to the cell will continue to occur and nutrient supply is greatly limited in the deep biosphere. Nevertheless, endospores might persist over long timespans in nutrient limited sedimentary environments.

The deeply buried coalbeds off Shimokita explored during the Integrated Ocean Drilling Program (IODP) Expedition 337 represent a very unique environment to investigate the boundaries of microbial life in deep subsurface sediments. Several layers of thermally immature lignites were buried sub-adjacent to marine sediments and contain energy-rich potential substrates that may create oases of life in the deep subseafloor (Fry et al., 2009; Glombitza et al., 2009b). Microbial life discovered in the Shimokita coalbeds consists mainly of persisters of microbes that initially inhabited the ancient forest soil and that have survived more than 20 Ma of burial (Inagaki et al., 2015). Cell numbers are extremely low in these deep sediments (1–10 cells cm^−3^) but are elevated up to ~1000 cells cm^−3^ in the coal bearing horizons. The increased temperature of >45°C most likely causes difficulties for microbial survival as DNA depurination and amino acid racemization reactions increase dramatically at these temperatures (Inagaki et al., 2015; Lever et al., 2015). The increased abundance of potential substrates in the organic matter-rich lithologies might, however, provide a large energy reservoir to sustain microbial life operating at its limits. Little is known about the variety of *in-situ* metabolic processes occurring in these sediments. Based on high concentrations of methane with an isotopic signature that indicates a biogenic origin, methanogenesis is an important metabolic process, however, the potentially huge availability and variety of electron donors might also enable other biotic processes. In this study, we investigated sulfate reduction by measurements of potential sulfate reduction rates (pSRR) in the Shimokita coalbeds using the radio-tracer (^35^${\text{SO}}_{4}^{2-}$) incubation technique (Jørgensen, 1978; Røy et al., 2014). The aim of this study was to reveal whether sulfate-reducing microorganisms were able to persist in the deeply buried, sulfate-depleted sediments over several millions of years of burial. In this context, we discuss the availability of potential electron donors (volatile fatty acids, methane, hydrogen), as well as the electron acceptor sulfate using the concentrations and isotopic composition of solid phase sulfur fractions, in particular of pyrite, in these deep coal-bearing sediments.

## Materials and methods

### Study area and sample material

#### IODP site C0020

**Figure 1:**
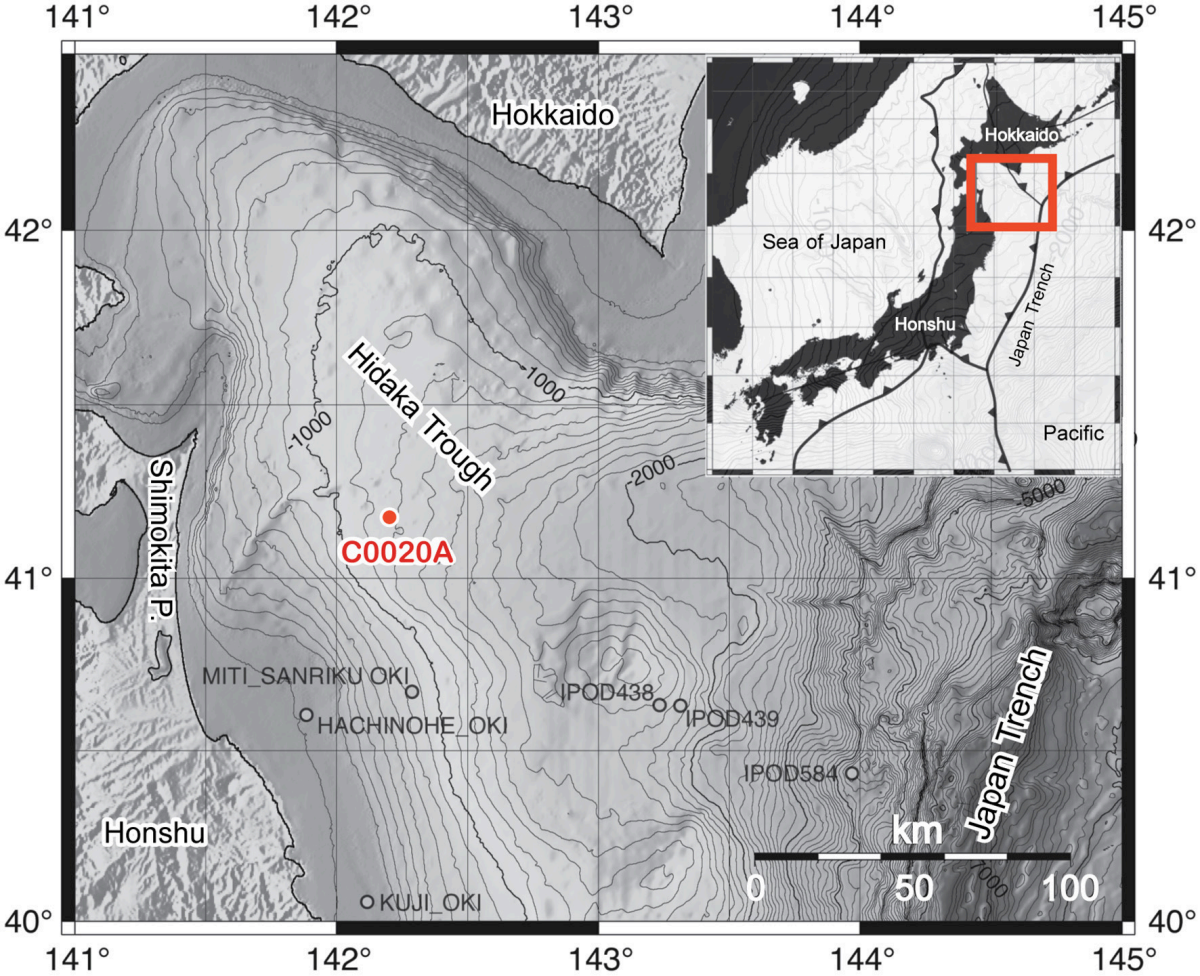
**Bathymetric map of the Hidaka Trough, bordered by the Japanese islands Honshu and Hokkaido and the Japan Trench, including the location of the IODP Expedition 337 Site C0020 Hole A (C0020A) and several previous drill holes in the area**. The insert shows the location of the plate boundaries around the Japanese islands and the exact location of the insert map. The maps are modified from Gross et al. (2015) with permission of the authors.

IODP Site C0020 is located ca. 80 km west off the coast of the Shimokita Peninsula, Japan (41°10.5983′N, 28 142°12.0328′E) at a water depth of 1180 m. The study site is located in a forarc basin, the Hidaka Trough, formed by the subduction of the Pacific plate under the Okhotsk plate (Maruyama et al., 1997). The Hidaka Trough extends between the Islands Hokkaido and Honshu and southeastwards to the Japan Trench (Figure 1). In this area, Cenozoic sedimentary and volcanic deposits overlie Triassic to Early Cretaceous rocks and granites (Inagaki et al., 2012, 2016). Coal-bearing horizons were confirmed by natural gas exploration drilling at the MITI Sanriku-oki site located ~50 km south of Site C0020 (Osawa et al., 2002).

During the *Chikyu* Shakedown Cruise CK06-06 in 2006, a riser-pilot hole was drilled down to 647 meters below seafloor (mbsf) and casing was installed up to 511 mbsf (Aioke, 2007). During this cruise, 365 m of sediment cores were recovered by *Chikyu'*s non-riser drilling, comprising diatomaceous silty clays that were intercalated with sand and tephra layers. The Site (JAMSTEC C9001) was later renamed to C0020 for the IODP drilling operation. Seismic profiles around Site C0020 suggested the presence of methane hydrates in sediments down to ~360 mbsf and a strong flux of free gas from deeper reservoirs (Inagaki et al., 2012).

IODP Expedition 337 (July–September 2012) reentered the hole and extended it to a final depth of 2466 mbsf and thereby pioneered riser-drilling technology in the deep-biosphere research through scientific ocean drilling (Inagaki et al., 2013, 2016). The bottom core from Hole C0020A is currently the deepest sample in the history of scientific ocean drilling, extending the previous depth record (ODP Leg 148, hole 504B, Alt et al., 1993) by 355 m. During Expedition 337, four lithostratigraphic units were defined on the basis of cuttings, cores, X-ray CT scans of the cores, and wireline logging data (Inagaki et al., 2012; Gross et al., 2015). Unit I (647–1256.5 mbsf) consist of primarily diatom-bearing silty clay of Pliocene age and results from sedimentation in an offshore marine environment in a cool-water continental shelf succession with elevated marine productivity. Unit II (1256.5–1826.4 mbsf) consists of shales with several intervals of siltstone and sandstone. The sediments are of early to middle Miocene age and were deposited mainly in a shallow marine environment, whereas the upper part of this unit was deposited in deeper water of a shelf area. Unit III (1826.5–2046.5 mbsf) contains coarse- to fine-grained clastic deposits with 13 imbedded lignite layers. Mostly, these seams are about 1 m thick, except for two seams with a thickness of 3.5 and 7.3 m. The thermal maturity of the lignites is 0.37–0.43% vitrinite reflectance (R_0_; Gross et al., 2015). The sediments in Unit III were deposited during early to middle Miocene in a near-shore environment with tidal flats and channels and wetlands (back marshes and swamps). Unit IV (2046.5–2466 mbsf) consist of shales, siltstones, sandstones, and a single small, ~1 m thick lignite layer at the base with a maturity of 0.47% R_0_. The pollen flora suggests a maximum age of late Oligocene for the base of Unit IV (Inagaki et al., 2012).

#### Samples for potential sulfate reduction rate measurements and hydrogenase enzyme activity in the deep sediment cores (IODP expedition 337)

Samples for pSRR and hydrogenase enzyme activity measurements were taken from whole round core (WRC) pieces of ~5–10 cm length taken from the core section immediately after retrieval on board the *D/V Chikyu* and after a quick CT scan of the core sections. The CT images were used to identify undisturbed core intervals where no fractures were found. In such core intervals, contamination by drilling fluid was expected to be only minor. A total of 27 WRC samples were collected between 1276.75 and 2456.72 mbsf (Units II–IV), comprising different lithologies including fine sands, sandstones, siltstones, silty clays, shales, and lignites (Table 1). The WRC pieces were vacuum sealed in gas-tight foil bags (ESCAL®) after flushing with N_2_ and stored at 4°C until further treatment usually within 1–5 h. For sub-sampling the sealed WRC pieces were transferred into an anoxic glove box. The outer centimeter of the core was removed with a sterile spatula to remove layers where drilling fluid might have penetrated in. The cleaned WRC was cracked into pieces and powdered in a sterile titanium mortar if necessary (i.e., for consolidated or hard core material) and ~5 cm^3^ of the powder was filled in a baked and pre-weighed 10 mL headspace vial and sealed with a rubber stopper and crimp cap. Four sub-samples were prepared from each WRC. The samples were immediately used for the incubation experiments to measure pSRR.

**Table 1:** **Characteristics (core and section numbers, depth, lithology, lithological unit) and mean potential sulfate reduction rate (pSRR) of all replicates including relative standard deviation (RSD), indicated exclusion of samples with RSD>50% and resulting mean pSRR and RSD for conservatively selected sample replicates**.

**Core-section**	**Depth [mbsf]**	**Sample lithology**	**Unit**	**All replicates**		**Excluded samples**	**Conservative replicates**	
				**Mean pSRR [pmol cm^−3^ d^−1^]**	**RSD [%]**		**Mean pSRR [pmol cm^−3^ d^−1^]**	**RSD [%]**
1R-1	1276.75	Fine sand	II	0.68	110	All replicates	–	–
2R-2	1287.87	Sandstone	II	110	158	All replicates	–	–
3R-2	1371.94	Siltstone	II	1.52	171	All replicates	–	–
6R-1	1495.05	Sandstone	II	0.08	121	All replicates	–	–
8L-5	1607.26	Shale	II	0.02	65	All replicates	–	–
9R-1	1625.56	Sandstone	II	0.03	42	No	0.03	42
10R-1	1630.16	Siltstone	II	0.05	87	1 outlier	0.03	21
11R-1	1738.80	Sandstone	II	0.06	40	No	0.06	40
13R-4	1760.49	Siltstone	II	0.02	39	No	0.02	39
14R-2	1822.41	Siltstone	II	0.04	48	No	0.04	48
15R-3	1921.98	Lignite	III	0.04	49	No	0.04	49
15R-6	1924.13	Shale	III	0.07	41	No	0.07	41
16R-3	1930.42	Fine sand	III	1.31	80	All replicates	–	–
18R-1	1945.71	Lignite	III	1.15	64	1 outlier	0.81	42
19R-1	1950.04	Sandstone	III	1.47	157	1 outlier	0.32	49
20R-5	1965.11	Shale	III	1.75	19	No	1.75	19
23R-3	1984.25	Siltstone	III	1.17	67	1 outlier	0.79	36
25R-2	1997.54	Lignite	III	0.56	42	No	0.56	42
25R-3	1998.75	Silty clay	III	1.10	45	No	1.10	45
26R-4	2113.51	Shale	IV	1.02	40	No	1.02	40
27R-1	2200.91	Shale	IV	0.64	37	No	0.64	37
28R-4	2304.83	Siltstone	IV	0.36	27	No	0.36	27
28R-5	2305.34	Siltstone	IV	0.31	12	No	0.31	12
29R-5	2405.52	Siltstone	IV	0.23	36	No	0.23	36
30R-2	2447.61	Lignite	IV	0.35	13	No	0.35	13
30R-3	2449.43	Shale	IV	0.12	52	1 outlier	0.10	44
32R-1	2456.72	Shale	IV	0.18	44	No	0.18	44

Additionally, a slice from each cleaned WRC (~20 cm^3^ intact core material) was double-packed in gas-tight plastic foil bags (ESCAL®, Mitsubishi Gas Chemical Co. Inc., Tokyo), flushed 3 times with N_2_, sealed under vacuum and stored frozen at −80°C. The hydrogenase enzyme essay only measures the activity of present, intact enzymes. Thus, in contrast to microbial activity measurements, it does not require metabolically active cells. However, immediate deep-freezing is important to preserve the enzymes in the sediment and prevent activity loss by oxidation (see Adhikari et al., 2016 for details). These samples were shipped deep-frozen to the home laboratory for shore-based hydrogenase enzyme activity measurements (Supplementary Table 2).

#### Samples for potential sulfate reduction rate measurements in the upper 350 mbsf (CK06-06, D/V Chikyu Shakedown Cruise)

All sediment samples of the C9001 Hole C core were sub-sampled onboard *D/V Chikyu* during the Shakedown Cruise CK06-06 in 2006. 5 cm^3^ of sediment were taken from the center of the drill cores by a tip-cut sterilized syringe in lamina-flow clean bench, immediately sealed with butyl rubber cap, and stored in an anaerobic chamber with an AnaeroPack (Mitsubishi Gas Chemical Co. Inc.) oxygen-removal reagent filled with nitrogen at 4°C. The sample preparation was performed in the microbiology laboratory onboard the *Chikyu*.

#### Samples for analysis of sediment sulfur fraction and isotopic composition

Sediment samples from nearly all cores covering the three lithological units were analyzed for reduced sulfide species (Supplementary Table 2). A total of 48 sample splits were collected from WRCs designated for microbiology analysis (Inagaki et al., 2013). The splits were taken in an anoxic glove box and sealed in gas-tight bags under N_2_ atmosphere after contamination screening based on measured concentrations of perfluoromethylcyclohexane, a perfluorocarbon compound, which had been added to drilling fluid as a chemical tracer (Inagaki et al., 2013). The samples were stored (and shipped) frozen until further processing for geochemical analysis.

### Methods

#### Potential sulfate reduction rates in the deep sediments

pSRR were measured by incubation of ^35^S labeled sulfate tracer in sediment slurries (Jørgensen, 1978). Slurries were prepared inside an anoxic glove box by adding 5 mL of sterile, anoxic, artificial seawater medium to each sample. The medium was prepared from 25 g L^−1^ NaCl, 5 g L^−1^ MgCl_2_ × 6 H_2_O, 0.5 g L^−1^ KCl, 0.2 g L^−1^ KH_2_PO_4_, 0.25 g L^−1^ NH_4_Cl, 0.15 g L^−1^ CaCl_2_ × 2 H_2_O, 2.5 g NaHCO_3_, and 1 mL of a 10 g L^−1^ solution of Resazurin as oxygen indicator. The pH was adjusted to 7.5 by adding NaOH solution (1 mol L^−1^) and oxygen was removed by the addition of a few drops of NaS_2_ solution (10 g L^−1^) until the indicator became colorless. Additionally, the medium was amended with 1 mmol L^−1^ Na_2_SO_4_. To each sample, 30 μL of carrier-free sulfate tracer (3.7 MBq) were added with Hamilton® gas tight syringes through the septum. In 2 of the 4 replicates 10 mL of pure CH_4_ was added via a syringe to the headspace, which increased the pressure inside the vial to ~2 bar. The samples were shaken and incubated for 10 days at 3 different temperatures to approximate *in-situ* temperatures. Samples between 1276.75 and 1500 mbsf were incubated at 25°C, samples between 1500 and 1980 mbsf were incubated at 35°C and samples between 1980 and 2456.75 mbsf were incubated at 45°C. To terminate the incubations, 3 mL of a 20% (w/v) zinc acetate solution were injected through the septum and the vial was shaken. Subsequently, the vial was opened and the content was transferred into a 50 ml Falcon® tube containing 7 mL 20% (w/v) zinc acetate solution and frozen at −20°C until further analysis in the home laboratory.

To calculate the sulfate reduction rates (SRR), the total reduced inorganic sulfur (TRIS) was extracted from the sediment by a cold chromium distillation procedure (Kallmeyer et al., 2004) following the modifications and recommendations made by Røy et al. (2014). Na_2_S (200 μL, 0.5 mol L^−1^) was added to the reaction flask as a sulfide carrier. At the end of the distillation, the distillate recovered in the zinc acetate trap was transferred into a 20 ml scintillation vial with 15 mL scintillation liquid (Ecoscint XR, National diagnostics, Atlanta, GA, USA). The radioactivity of the total sulfur fraction (a_TOT_) and in the reduced sulfur fraction (a_TRIS_) was measured in a liquid scintillation counter (Packard Tri-Carb 2900 TR liquid scintillation analyzer). Samples were counted for 30 min. Blank samples, which were transferred to zinc acetate (20% w/v) before tracer injection, were used to determine the background. SRR were calculated according to Kallmeyer et al. (2004) (Equation 1):

(1) $$\begin{array}{r} SRR=\left[{SO}_{4}^{2-}\right]\times \Phi \times \frac{a_{TRIS}}{a_{TOT}}\times \frac{1}{t}\times 1.06 \end{array}$$

where [${SO}_{4}^{2-}$] is the sulfate concentration (1 mmol L^−1^), Φ is the porosity, a_TRIS_ is the radioactivity of the reduced sulfur fraction, a_TOT_ is the total sample radioactivity, t is the incubation time and 1.06 is the correction factor for the estimated microbial isotopic fractionation of sulfur during sulfate reduction (Jørgensen and Fenchel, 1974). Porosity data were measured onboard (Inagaki et al., 2015; Tanikawa et al., 2016). Measurements of *in-situ* sulfate concentrations were disturbed by contamination of pore water samples by drilling fluid (Inagaki et al., 2013). Based on the porewater profile at shallow depths (Tomaru et al., 2009) and the abundant methane (Inagaki et al., 2015) we assume that sulfate in the deep sediments is depleted or only present in trace amounts.

#### Potential sulfate reduction rates in shallow (<350 mbsf) sediments

pSRR were determined by incubations of sediment slurries with ^35^S-labeled sulfate, similar as described above. In an anaerobic glove box, 3 cm^3^ of a 5 cm^3^ mini-core sample stored at 4°C in anaerobic chamber were transferred into a 15 ml test tube (1 cm^3^ at both ends was removed) and 10 ml of sterilized anoxic artificial seawater containing 10 mmol L^−1^ sulfate was added. The test tubes were capped with a rubber stopper, and 500 kBq of ^35^S-labeled sulfate was injected. After 100 days of incubation at 5°C, the reaction was stopped by adding 20 ml of zinc acetate (20% w/v). Reduced sulfur compounds were distillated by following the cold chromium distillation method (Kallmeyer et al., 2004). After the distillation, 3 ml of the solution in the zinc acetate trap containing the TRIS fraction was mixed with 12 ml Hionic-Fluor liquid scintillation cocktail (PerkinElmer) and the radio-activities of the total reduced sulfur fraction (a_TRIS_) and the total sulfur fraction (a_TOT_) were determined with a Tri-Carb^TM^ 2900TR Liquid Scintillation Analyzer (PerkinElmer). pSRR were calculated as described above.

#### Solid phase sulfur species and isotopic composition

The samples were sequentially analyzed using ~2–3 g frozen wet sediment. For determination of acid volatile sulfide (AVS) and chromium reducible sulfur (CRS) the samples were treated with a two-step acid Cr(II) method (Canfield et al., 1986; Fossing and Jørgensen, 1989) under anoxic conditions. The released sulfide was trapped in a 5% (w/v) zinc acetate solution for each step (AVS and CRS). Aliquots of the sulfide precipitate as ZnS were analyzed upon dilution by the methylene blue method (Cline, 1969). The sulfide concentrations are reported on a dry weight basis adjusting for sample wet weight.

For sulfur isotope (δ^34^S) analysis, the trap-solutions were centrifuged, the supernatant decanted, and the precipitated rinsed with oxygen-free double-ionized water. ZnS precipitates were converted to Ag_2_S by treatment with 3% (w/v) AgNO_3_ and subsequent washing with NH_4_OH to remove colloidal silver. The cleaned Ag_2_S precipitate was dried at < 40°C. All sulfur isotope samples were weight into tin capsules and V_2_O_5_ was added to ensure complete combustion. The samples were analyzed at Indiana University-Purdue University Indianapolis using a Costech Elemental Analyzer connected under continuous flow to a Thermo Delta V Plus stable isotope ratio mass spectrometer (EA-IRMS). All sulfur isotope measurements were calibrated with reference materials IAEA S1 (δ^34^S = −0.30‰), IAEA S2 (δ^34^S = −22.65‰), and IAEA S3 (δ^34^S = −32.50‰) and the precision (1σ) based on repeated measurement of each standard (*n* = 5) was better than 0.2‰.

Sample δ^34^S values were reported relative to Vienna Canyon Diabolo Troilite (VCDT) according to Equation (2):

(2) $$\delta^{34}S=\left[\frac{{{(}^{34}S{/}^{32}S)}_{sample}}{{{(}^{34}S{/}^{32}S)}_{VCDT}}-1\right]\;\times \;1000$$

#### Hydrogen utilization potential by hydrogenase enzymes in the deep sediments

A tritium–based hydrogenase enzyme assay was used to measure potential oxidation rates of hydrogen (Soffientino et al., 2009; Adhikari et al., 2016) at the University of Bremen. In short, three replicates and a killed control were prepared from frozen sediment for the incubation experiment using anoxic autoclaved synthetic seawater medium. The slurries were incubated with tritium (20% H_2_/N_2_) at room temperature (~25°C) under continuous shaking (250 rpm). Five subsamples were collected after 0.5, 1, 2, 3, and 4 h of incubation. Unreacted tritium was removed by flushing with N_2_ and the suspended particles were separated by centrifugation. 100 μL of the supernatant was mixed with 4 mL liquid scintillation fluid (Perkin Elmer Ultima Gold™ LLT) and the radioactivity was measured in a liquid scintillation analyzer (Perkin Elmer TriCarb® TR2810). The increase in radioactivity over time was used as a measure of the potential hydrogenase activity (see (Adhikari et al., 2016) for details).

#### X-ray computer tomography

X-ray computer tomography (CT) images were generated for each core section using a GE Yokogawa Medical Systems LightSpeed Ultra 16 (GE Healthcare, 2006) on board the *Chikyu* during Expedition 337 (Inagaki et al., 2013). Analytical standards included air (CT number = −1000), water (CT number = 0), and aluminum (2477 < CT number < 2487) in an acrylic core mock-up. For detailed description see Inagaki et al. (2013).

## Results

### Potential sulfate reduction rates in the shallow (<350 mbsf) sediments

**Figure 2:**
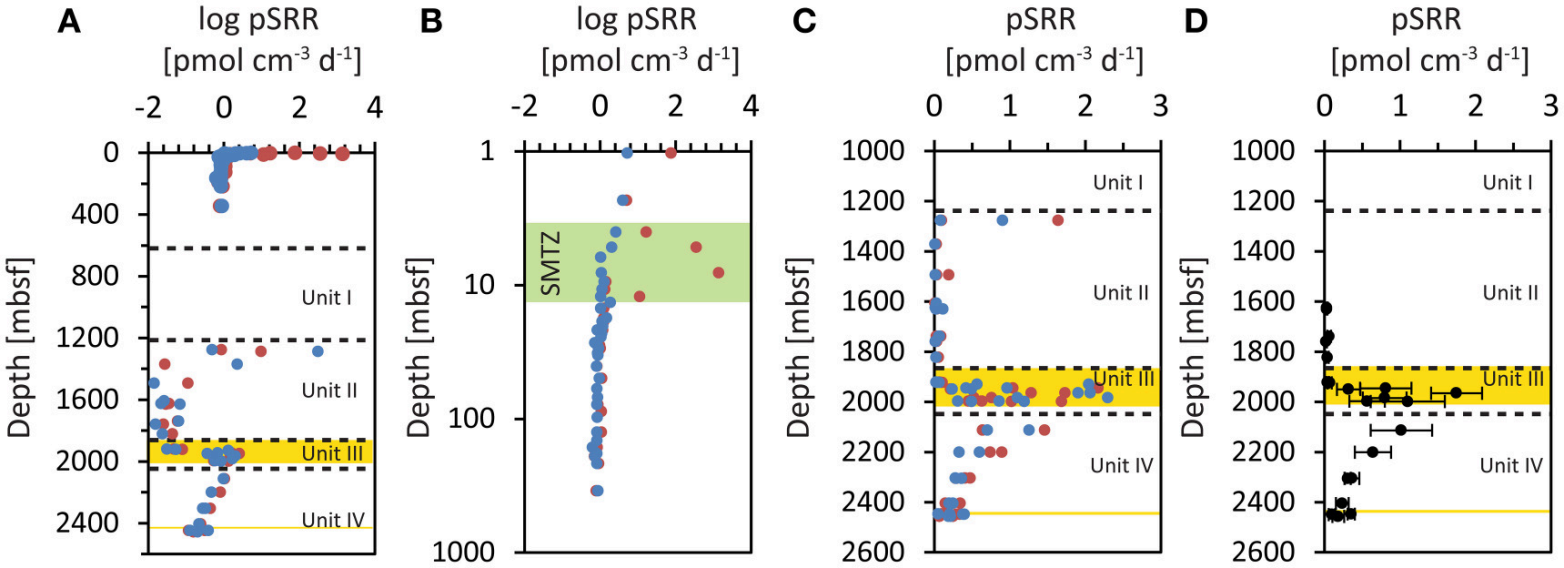
**Potential sulfate reduction rates (pSRR) measured in incubations of sediment slurries at IODP Site C0020**. Incubations with methane amended to the headspace are shown in red, incubations without methane amendment to the headspace are shown in blue. **(A)** Mean pSRR incubated with ^35^S labeled sulfate. **(B)** Mean pSRR in the upper 350 mbsf, incubated during CK06-06 (note the log depth scale). The green zone highlights the sulfate methane transition zone (SMTZ; (Kobayashi et al., 2008; Tomaru et al., 2009)). **(C)** pSRR of individual sample replicates from incubations during IODP Expedition 337 (note only pSRR values below 3 pmol cm^−3^ d^−1^ are plotted). **(D)** Mean pSRR of all replicates (with and without methane amendment) from incubations during the IODP Expedition 337 after conservative exclusion of potentially contaminated samples (see text). The error bars represent ± one standard deviation.

pSRR measured during the *Chikyu* Shakedown Cruise CK06-06 in 2006 are shown in Figures 2A,B. Rates measured in slurries with the amendment of methane to the headspace are shown in red, incubations without methane amendment in blue. pSRR decreased with depth from ~5 pmol cm^−3^ d^−1^ in the upper 1–2 mbsf down to 0.7 pmol cm^−3^ d^−1^ in the deepest samples down to 346.28 mbsf. Samples incubated with the amendment of methane to the headspace showed an intermittent increase in pSRR between 4 and 12 mbsf with a maximum of 1380 pmol cm^−3^ d^−1^ at 8.1 mbsf (Figure 2B). This coincided with the depletion of porewater sulfate (Tomaru et al., 2009) and the onset of methane in the sediments (Kobayashi et al., 2008), and indicates increased rates of anaerobic oxidation of methane (AOM) in the SMTZ. Below 12 mbsf, the measured pSRR were indistinguishable in incubations with and without the amendment of methane.

### Potential sulfate reduction rates in the deep sediments

pSRR could be determined in nearly all sample replicates from the cores between 1276.75 and 2456.72 mbsf (Table 1). There was no evident difference between incubations with (red symbols) and without (blue symbols) the amendment of methane to the headspace of the incubation vials (Figures 2A,C). Thus, we can consider all incubation slurries of each WRC as replicates. The mean values of all four replicates of the WRCs range between 0.02 pmol cm^−3^ d^−1^ and 1.75 pmol cm^−3^ d^−1^ with exception of core 2R-2, which was two orders of magnitude higher. For some samples, the relative standard deviation (RSD) of the four replicates was extremely high, e.g., 171 and 158% for cores 3R-2 and 2R-2, respectively (Table 1), which indicates broad differences between replicates. However, the relative standard deviation of the method is usually around 10–20% (Røy et al., 2014) whereas in some low activity environments deviations up to ~50% were reported (Nickel et al., 2012). In particular, outliers with very high values were measured in cores 2R-2 (12 and 309 pmol cm^−3^ d^−1^) and 3R-2 (5 pmol cm^−3^ d^−1^), (Figure 2A). However, at the spatial scale (~20 cm^3^ of inner core material) of which the individual replicates were sampled, a rather homogeneous distribution of microorganisms can be expected if the sample lithology also appeared homogenous. Thus, the individual technical replicates should result in similar rate measurements. A broad scattering within the four replicates (Figure 2C) and especially outliers with unusual high rate numbers could therefore indicate potential contamination (e.g., by intrusion of drilling fluid into micro-fractures). To carefully exclude such potentially contaminated samples, we made a conservative selection of samples on the basis of the RSD of the four replicates. We excluded the upper outlier of the four replicates if the RSD was >50%. For 5 cores (10R-1, 18R-1, 19R-1, 23R-3, and 30R-3), this procedure resulted in new mean values of the remaining three technical replicates with RSD meeting the criterion. For six other cores (1R-1, 2R-2, 3R-2, 6R-1, 8L.5, and 16R-3) this procedure did not lead to mean values that meet the criterion. These cores were fully excluded from the conservative selection of samples (Table 1). The mean values of the pSRR from the conservatively selected samples contained replicates with an RSD < 50% only (Figure 2C, Table 1). In this data-set, the scale of variability observed between the lithological units was far greater than the internal variability observed for each depth. In cores from Unit II, pSRR values are consistently low, between 0.02 pmol cm^−3^ d^−1^ and 0.07 pmol cm^−3^ d^−1^. In the Unit III containing the embedded lignite layers, the pSRR were elevated by one to two orders of magnitude, with values up to 1.75 pmol cm^−3^ d^−1^. The highest rate was measured in core 20R-5 (1.75 pmol cm^−3^ d^−1^) representing a shale while the shale sample from core 15R-6 showed only 0.07 pmol cm^−3^ d^−1^ (Table 1). Similar, pSRR in the lignite sample of core 18R-1 (1.15 pmol cm^−3^ d^−1^) was higher than in the lignite layer ~50 m deeper in core 25R-2 (0.56 pmol cm^−3^ d^−1^). This shows that high rates are not necessarily tied to a specific lithology and conclusions about a certain lithology representing a “hot-spot” for sulfate reducers cannot be drawn. This might also be concealed by our relatively low sampling resolution, as e.g., heterogeneity in cell distribution even over a small scale of a few mm were recently reported in lacustrine sediments (Kallmeyer et al., 2015). Nevertheless, the overall elevated pSRR in Unit III are clearly visible in Figure 2. In Unit IV, the pSRRs decreased continuously with depth from 1.02 pmol cm^−3^ d^−1^ to 0.12 pmol cm^−3^ d^−1^ but in the deepest sample (32R-1, 2456.72 mbsf) they were still approximately one order of magnitude higher than in core samples from Unit II.

### Solid phase sulfur fractions and isotopic composition in the coal beds

**Figure 3:**
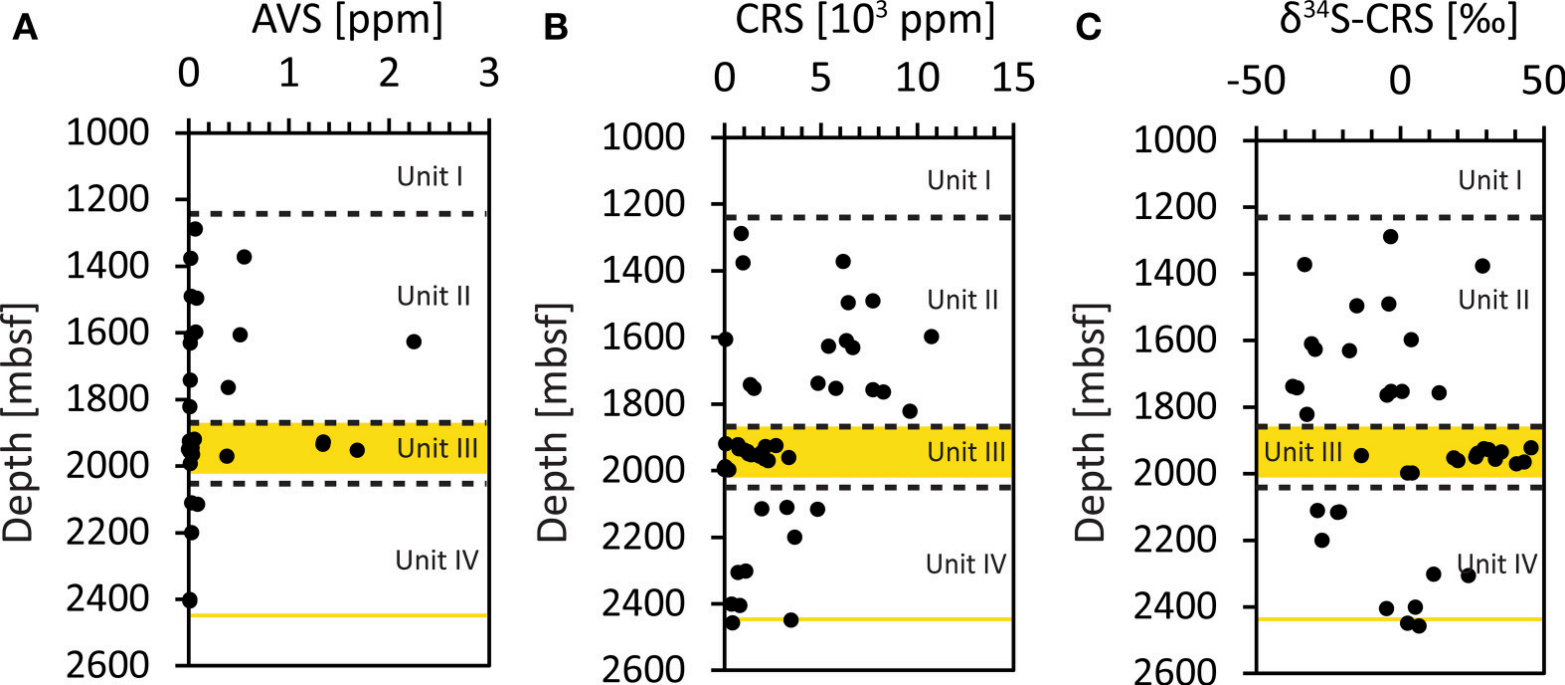
**Concentrations of solid phase sulfur fractions. (A)** AVS (acid-volatile sulfide) fraction, **(B)** CRS (chromium-reducible sulfur) fraction. **(C)** Isotopic composition (δ^34^S) of the CRS fraction.

The amount of the acid-volatile sulfide (AVS) fraction was very low in all investigated samples, with the majority of samples below 0.6 ppm. In one sample from Unit II (9R-4, 1628.45 mbsf) and in three samples from Unit III (16R-2, 1929.6 mbsf; 17R-1, 1936.53 mbsf and 19R-5, 1954.53 mbsf), the values were slightly elevated, between 1.35 and 2.25 ppm (Figure 3A, Supplementary Table 2). The amount of AVS, however, was too low for stable sulfur isotope analysis. The sediments display a broad range (3.7–10753 ppm) of the chromium-reducible sulfur (CRS), which generally decreases with increasing sediment depth (Figure 3B, Supplementary Table 2). However, samples from Unit III had consistently low values (< 2681 ppm) and comprised the samples with the lowermost CRS amounts (3.7–198 ppm, cores 23R, 24R and 25R). The δ^34^S of CRS varied over a broad range from −33.1 to +45.6‰ (Figure 3C, Supplementary Table 2). The lowest δ^34^S values were found in Unit II with a median of −9.8‰. The coal bearing Unit III contained CRS with higher δ^34^S (median: +26.9‰) and included the samples with the most pronounced ^34^S-enrichment, in cores 15R-5 (1923.74 mbsf, +45.6‰), 20R-7 (1966.38 mbsf, +43.3‰), and 21R-4 (1971.78 mbsf, +40.5‰), these samples mainly comprised coals and coaly shales. For some of the coal samples (i.e., in cores 22R, 23R, and 24R) the CRS content was too low to determine the isotopic composition. In Unit IV, the δ^34^S values in the upper sediment layers (2100–2200 mbsf) have significantly lower δ^34^S values compared to samples deeper in the unit (2300–2448 mbsf; Figure 3C).

### Potential for hydrogen utilization in the deep sediments

**Figure 4:**
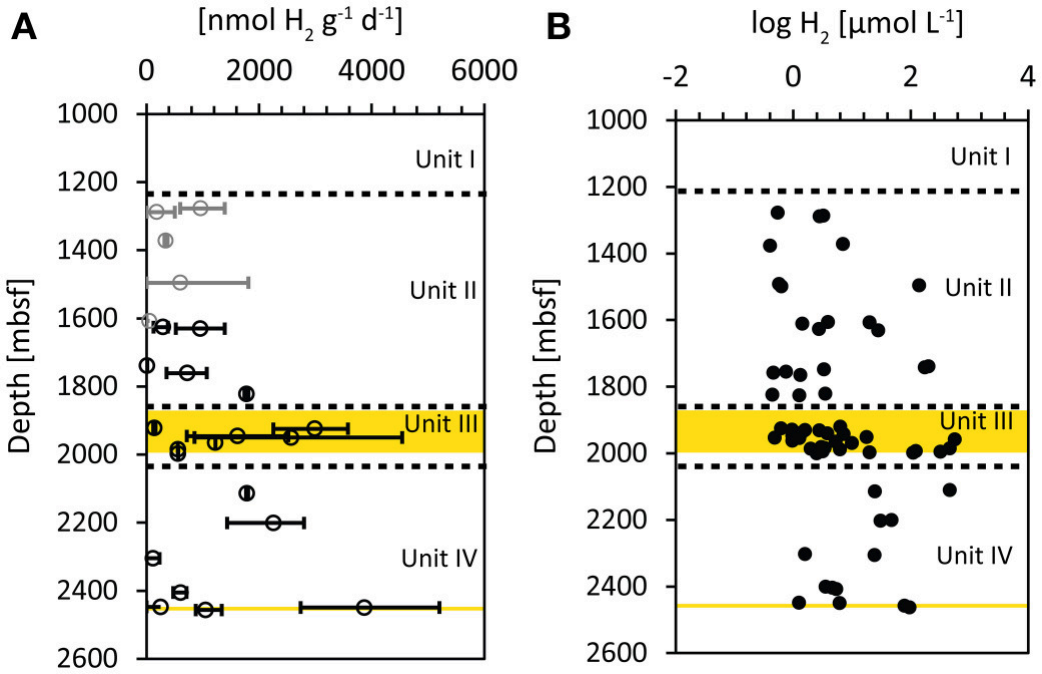
**(A)** Potential hydrogen oxidation rates measured by incubations with tritium gas (^3^H_2_). The error bars represent minimum and maximum values of the sample replicates, circles represent average values. Gray symbols represent samples from WRCs that were excluded from the conservative selected pSRR data. **(B)** Log of hydrogen concentrations in the sediment pore water, determined by the extraction method (Inagaki et al., 2015). Yellow areas represent zones with imbedded lignite layers.

The potential hydrogen oxidation rates (Figure 4A) could be measured in all cores with exception of 16R-3, 25R-3, and 28R-5. The measured values ranged between 13 nmol H_2_ g^−1^ d^−1^ (11R-1) and 5200 nmol H_2_ g^−1^ d^−1^ (30R-2; Supplementary Table 1). As a result of the diversity of hydrogenase enzymes with different activities utilized by various anaerobic microorganisms, the measured rates of hydrogen oxidation cannot be used to infer *in-situ* rates of metabolic processes in the sediments (Adhikari et al., 2016). However, they are suggested to indicate the presence of metabolically active cells utilizing hydrogen in sediments (Soffientino et al., 2009). Recently it was reported that sulfate reducers actively express hydrogenases in subseafloor sediments (Orsi et al., 2016). Because the samples used for the tritium assay were not taken from the exact same splits as the samples for pSRR measurements, we did not exclude measured hydrogen oxidation rates on the basis of excluded pSRR replicates. However, the samples from the WRCs that were excluded in pSRR data are indicated by gray symbols (Figure 4A). The measured rates in Unit II are all less than 2000 nmol H_2_ g^−1^ d^−1^, with mean values around 600 nmol H_2_ g^−1^ d^−1^. Highest values were found in the coal bearing Unit III in samples 15R-6 (1924.13 msbf, max. rate: 3579 nmol H_2_ g^−1^ d^−1^) and 19R-1 (1950.04, max rate: 4538 nmol H_2_ g^−1^ d^−1^), as well as in the WRC sample adjacent to the deep lignite layer (ca. 2447 mbsf) in sample 30R-3 (2449.43 mbsf, max. rate: 5201 nmol H_2_ g^−1^ d^−1^) but not in the lignite itself.

## Discussion

### Potential sulfate reduction rates in the deep coalbeds

The highest rates of sulfate reduction are typically found in near-surface sediments (the upper 10's of centimeters up to a meter sediment depth) and decrease with depth in an exponential manner (Jørgensen, 1977, 1982; Jørgensen and Parkes, 2010). This general pattern was also observed in the near-surface sediments at Site C0020 retrieved during the shakedown cruise CK 06-06 (Figure 2A). The significant increase of pSRR in incubations with amended methane between 4 and 12 mbsf indicates the occurrence of AOM in the SMTZ (Barnes and Goldberg, 1976; Iversen and Jørgensen, 1985; Caldwell et al., 2008). However, the detection of pSRR in the deep samples between 1500 and 2456.75 mbsf was surprising because pore water sulfate was likely depleted already in much shallower and younger sediments similar as observed in the shallow cores retrieved during the CK 06-06 cruise (Tomaru et al., 2009). Persisting sulfate reducers in the deep sediments off Shimokita thus would have remained metabolically intact over long periods of starvation at no or only trace amounts of sulfate. However, our finding shows that the deep sediments host microorganisms capable of sulfate reduction even after 20–25 million years of burial.

In addition to nutrient availability in the deep sediments (which is discussed further in the following sections) resulting in energetic limits of life in the deep biosphere (Lever et al., 2015; Jørgensen and Marshall, 2016), the sulfate reducing microorganisms buried in the deep sediments offshore Shimokita have to cope with physiological effects of increasing pressure and temperature. Increasing pressure affects e.g., motility, cell division, DNA replication, transcription, translation, or certain enzymatic reactions (Marietou and Bartlett, 2014) and increasing temperature has recently been shown to increase damage of cell walls and DNA by protein racemization and DNA depurination reactions (Lever et al., 2015). In a recent study, sulfate reducers isolated from deep and hot (~60°C, 30 MPa) sediments at the Juan de Fuca Ridge showed adaptation to the increased pressure and temperature e.g., by exchanged membrane lipid composition and an increase of their optimum growth temperature (Fichtel et al., 2015).

The measured potential rates in the deep samples (0.02–1.75 pmol cm^−3^ d^−1^) were among the lowest sulfate reduction rates measured so far (around 0.1 pmol cm^−3^ d^−1^; Jørgensen and Marshall (2016), Parkes et al. (1990), and references therein). The detectability of such low sulfate reduction rates was a result of our optimized long incubation times and the high initial radioactivity (3.7 MBq) incubated in each sample. The highest potential rate, observed in the Unit III that contained the coal beds was at the level of the lowest rates measured in the deepest samples from the CK06-06 cruise at ~350 mbsf (Figure 2A). It is important to note that the rates measured in our incubation experiments are not *in-situ* rates. It has been demonstrated that making sediment slurries affects the measured rates and the results differ from incubations of sub-cores where the original sediment structure remains undisturbed (Jørgensen, 1978; Meier et al., 2000). An important factor is also that the sediment slurries were amended with 1 mmol L^−1^ sulfate to maintain a sufficient background concentration of sulfate to avoid immediate turnover of the carrier-free ^35^${\text{SO}}_{4}^{2-}$ tracer, which would result in false positive rates. This background concentration is a significant difference from the natural conditions in the deep sediments at Site C0020, where pore water sulfate is assumed to be depleted. The chosen incubation time (10 days) was rather long and in combination with the elevated sulfate concentration in the slurries, it is likely that sulfate-reducers have been stimulated during incubation. The sulfate-reducers might have been present only as spores in these sediments and the incubation experiment might have triggered germination. It has been shown previously that spores are frequently abundant in marine sediments with numbers equal to those of vegetative cells (Lomstein et al., 2012). In fact, spore-like particles were also detected by microscopic observations in the deep sediment samples (Inagaki et al., 2015). However, even if the sulfate reducers have only survived in a dormant, spore-like state, our results indicate the capacity for microbial sulfate reduction in the very deep sediments and provide evidence for the deepest occurrence of sulfate reducers in sediments to date.

### Availability of potential electron donors

It is remarkable that the pSRR were elevated in the Unit III where the coal beds are located. It suggests that a larger number of sulfate reducers have survived in that particular, organic matter-rich sediment zone. The most important electron donors for sulfate reduction in anoxic sediments are organic acids such as volatile fatty acids (Sørensen et al., 1981; Christensen, 1984; Finke et al., 2006; Glombitza et al., 2015) and amino acids, which have been found to be actively cycled in the subsurface (Lomstein et al., 2012), especially by sulfate reducing bacteria (Parkes et al., 1994; Orsi et al., 2016). Volatile fatty acids are primary products of fermentation. As intermediates in the microbial mineralization of organic carbon in the sediments, their concentrations in the pore water especially in surface-near sulfate reducing sediments are usually relatively low, a result of their fast turnover (Sansone and Martens, 1982; Glombitza et al., 2014, 2015). In sediments where the turnover is slow or even prevented, volatile fatty acids can, however, accumulate and are found in higher concentrations (e.g., Martens, 1990; Wellsbury et al., 1997; Dhillon et al., 2005; Heuer et al., 2009). Substantial concentrations of volatile fatty acids have been found in water extracts of organic matter-rich sediments, in particular from coals and organic matter rich shales (Bou-Raad et al., 2000; Vieth et al., 2008; Zhu et al., 2015). Coals contain high amounts of macromolecular organic matter (Vandenbroucke and Largeau, 2007; Vu et al., 2009). Especially in low maturity coals, this macromolecular organic matter contains significant amounts of oxygen bearing functional groups, such as esters (Glombitza et al., 2009a, 2016) and ethers (Glombitza et al., 2011). In a previous study we showed that lignites can release acetate and formate from the macromolecular organic matter network during ongoing maturation in rates that are sufficient to sustain deep microbial life (Glombitza et al., 2009b). Such a constant supply of volatile fatty acids may provide electron donors for sulfate reduction in the deeply buried coalbeds.

In addition to organic acids, the sediments in Unit III are characterized by high amounts of methane (Inagaki et al., 2015). Methane can be oxidized by sulfate reduction in the deep sediments at Site C0020, in a similar fashion as it was observed by increased sulfate reduction rates in the SMTZ at 4–12 mbsf. The observation that no difference between incubations with and without amendment of methane to the headspace was found during incubations of the deep samples suggests that sulfate reduction at depth is not coupled to AOM. However, the coal bearing sediments have excess methane, and combined with the high adsorption affinity of hydrocarbons (including methane gas) in the microporous coal matrix (Clarkson and Bustin, 2000; Strapoc et al., 2007), splits from these samples might have led to methane-saturated incubation slurries in all incubations containing higher amounts of lignite. In this case, the pSRR from the coal-bearing Unit III might indeed reflect methane-driven sulfate reduction.

Another important electron donor utilized in microbial sulfate reduction is hydrogen (Lovley and Goodwin, 1988; Lovley and Chapelle, 1995; Hoehler et al., 1998), which was suggested to be of increased importance in deep sediments (Adhikari et al., 2016). Especially in deep methanogenic sediments (Schink, 1997) or in sediments lacking sufficient organic matter (Chapelle et al., 2002; Nealson et al., 2005) microbial processes were found to be dominated by hydrogen utilization. Usually hydrogen concentrations in shallow, relatively active sediments remain low as a result of the fast turnover rates (Hoehler et al., 2001). In contrast, at Site C0020 the concentrations of dissolved hydrogen below 1500 mbsf are relatively high with up to 500 μM (Inagaki et al., 2015; Figure 4B), pointing to very slow turnover rates and decoupling of hydrogen producing and consuming processes; the onset of such decoupling starts already in the shallower subsurface (Lin et al., 2012). As a result of the high hydrogen concentrations, Gibbs energy yield of hydrogenotrophic methanogenesis at Site C0020 is more negative (i.e., ~−100 kJ mol^−1^) than previously reported for deep sediments, and the combined carbon and hydrogen isotopic compositions of methane support its production by hydrogenotrophic methanogenesis (Inagaki et al., 2015). Gibbs energy yield for hydrogenotophic sulfate reduction should be highly negative as well, even at very low sulfate concentrations. However, Gibbs energy yield from sulfate reduction cannot be calculated due to the lack of pore water sulfide and sulfate concentrations. An indication of hydrogen utilization might be found in the hydrogen oxidation rates measured by the tritium assay (Figure 4A). The measured hydrogen oxidation rates reflect the activity of hydrogenases in the sediment (Adhikari et al., 2016). Hydrogenases form a diverse group of enzymes that are employed by microorganisms for both, hydrogen production (e.g., in fermentation) or hydrogen utilization. Thus, the increase in hydrogen oxidation rates observed in the Unit III might partly be the result of increased fermentation but at the same time the rates might indicate increased hydrogen consumption. The increase in hydrogenase enzyme activity in Unit III is not reflected by a significant increase in hydrogen concentrations. This suggests increased turnover of hydrogen in the coal-bearing unit. The fact that this increase was observed in the same depth interval as the increase in pSRR might point to potential hydrogenotrophic sulfate reduction. This is consistent with the observation that sulfate reducers were found to express hydrogenases in deeply buried sediments such as the carbon-monoxide dehydrogenase, as recently reported from the Peru Margin subseafloor (Orsi et al., 2016).

There are several potential electron donors for sulfate reduction (volatile fatty acids, amino acids methane, and hydrogen) that are presumably or evidently available in high amounts in the deep sediments at Site C0020. They are all released by biotic and abiotic degradation of organic matter and their high abundance relates to the high organic matter concentrations in the coals. Thus, sulfate reduction in the deep coalbed biosphere is obviously not electron donor limited.

### Availability of the electron acceptor sulfate

The availability of the electron acceptor sulfate is most likely the limiting factor for the occurrence of *in-situ* activity and presence of sulfate reducers at depth in Site C0020. Sulfate concentrations in the pore water obtained from the deeper samples of the CK06-06 cruise were between 0.1 and 0.3 mmol L^−1^, or below detection (Tomaru et al., 2009). Thus, the deep sediments drilled during IODP Expedition 337 are most likely almost sulfate-free or contain sulfate only in trace amounts. Sulfate concentrations measured in pore water samples from the deep drill cores were used as an indicator for a potential contamination of the pore water sample by seawater-containing drill mud (Inagaki et al., 2013). This approach was furthermore justified by the observation that the highest amounts of sulfate in pore water samples were found in samples, in which a contamination assay involving perfluorocarbon tracer indicated contamination (Lever et al., 2006; Inagaki et al., 2013).

**Figure 5:**
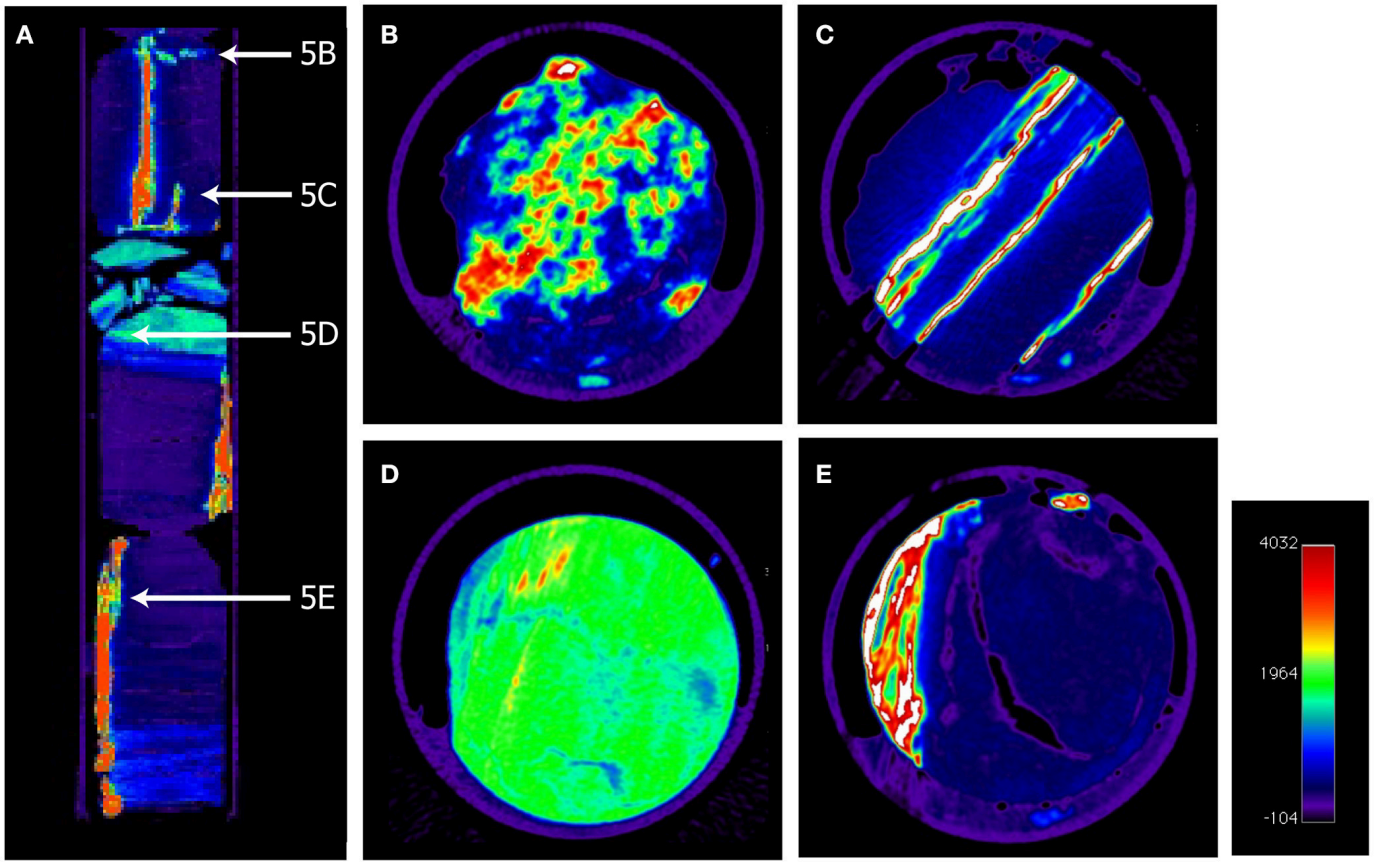
**Colored x-ray computer tomography pictures of a 34 cm long core segment of core 30R-2 showing vertical pyrite veins and pyrite granules**. **(A)** Vertical picture with indications of the positions of horizontal panels **(B–E)**. **(B)** Patchy pyrite precipitates, **(C)** Multiple vertical pyrite veins, **(D)** Mud-coal interface with small pyrites, **(E)** multiple pyrite veins in coal cavities.

Recently, it has been shown that sulfate reducers can persist also in methane bearing sediments below the SMTZ (Leloup et al., 2007, 2009) in sediments where trace amount of sulfate can be generated enabling a reductive sulfur cycling (Brunner et al., 2016). It was demonstrated that sulfate reduction occurs at low rates (0.2–1 pmol cm^−3^ d^−1^) also in the methane zone at constantly low background sulfate concentrations below 0.5 mmol L^−1^ (Holmkvist et al., 2011). It was speculated that the sulfate reduced in this depth was regenerated in a “cryptic sulfur cycle” by which the sulfide produced by microbial sulfate reduction is partly re-oxidized to sulfate in the presence of deeply buried Fe(III). In the sediments at Site C0020, glauconite was identified (Inagaki et al., 2013)—a Fe(III) source that can further be reduced to the final product pyrite via sulfide oxidation to thiosulfate by Fe(III) and subsequent disproportionation to sulfide and sulfate (e.g., Canfield and Thamdrup, 1994). The reduced Fe(II) can then form pyrite in the reaction with sulfide (Berner, 1970, 1984) whereas the sulfate is available for microbial reduction. By this process, part of the sulfate might be recycled and help to drive sulfate reduction in the deep sediments. As recently reported from Peru Margin sediments, the oxidation of sulfide via Fe(III) in the sediments may also be mediated by chemolithoautotrophic sulfur oxidizers (Orsi et al., 2016). It is interesting that the AVS fraction (e.g., the iron monosulfides) was at such low concentrations (Figure 3A), because in sulfide limited and iron rich sediments the metastable monosulfides are usually more abundant (Kasten et al., 1998). However, the *in-situ* sulfate reduction rates in the deep sediments off Shimokita are probably extremely low, considerably lower than the potential rates that we have measured in the slurry incubations. Such low rates might simply not significantly increase the AVS fraction and the slowly forming FeS may transform into pyrite at a similar pace. The major sulfur fraction in the sediments was the CRS fraction comprising mainly the pyrites (Figure 3B). It is remarkable that CRS concentrations in Unit III seem to be lower than in the overlaying sediments. This can, however, simply be a result of the accumulation of the pyrite in large granules and pyrite veins found in the coalbeds (Figure 5, and the movie clip showing a CT scan of core 30R-2, https://figshare.com/s/6ac50e6f31d5ce0b45f3). The analysis of the CRS fraction in bulk sediment samples might have captured only the low amounts of dispersed pyrite.

The distribution of sulfur isotopes revealed highly ^34^S-enriched sulfur in the CRS fraction (up to +45.6‰). This points to the formation of CRS in deeper sediments (Zaback and Pratt, 1992) from the reduction of isotopically enriched sulfate that had already experienced intense sulfate reduction in the sediment horizons overlaying the coal beds. In general, pyrites formed in coals can retain a large variability in δ^34^S ranging from approximately −15 to +27‰ (e.g., Price and Shieh, 1979). Smith and Batts (1974) described the occurrence of isotopically enriched pyrite in coalbed that were overlain by marine sediments and explained this by the reduction of sulfate that has diffused downwards and was already isotopically enriched during microbial reduction in the marine sediments. In sediments from the Black Sea, Jørgensen et al. (2004) showed that pyrite enriched in ^34^S (up to +33‰) can be formed during AOM of residual pore water sulfate with high δ^34^S (+43‰). Additionally, Canfield (2001) showed that the fractionation between sulfate and sulfide during microbial sulfate reduction diminishes at low sulfate concentrations. In our study, the isotopically heavy pyrite with δ^34^S > +45‰ in the Shimokita coalbeds might be explained by continuous sulfate reduction of an increasingly smaller, increasingly ^34^S-enriched sulfate reservoir. Although we cannot determine when exactly the pyrites in the Shimokita coalbed have been formed it is obvious that this has continued over long time scales and consequently also long after burial, as also suggested by the pSRR data.

The findings of sulfate reducers with DNA from ancestral cell lines in ~100 Ma old black shales led to the discussion of a “paleome,” a pool of ancient DNA and/or descendants preserved in the sediments by living microorganisms buried millions of years back in time which thus can provide insights into ancient forms of life (Inagaki et al., 2005). The finding of cells capable of sulfate reduction more than 20 Ma after burial in the sediments offshore Shimokita highlights the ability of sulfate reducers to persist in sediments over geological timespans even in sulfate-depleted environments. Although our data only capture a few tens of millions of years, this observation might support the “paleome” concept.

## Conclusion

pSRR was detected in the deep sediments at IODP Site C0020 off the Shimokita Peninsula, Japan, down to 2456 m below the ocean floor. The potential rates were extremely low but showed a significant increase in the coal-bearing horizon. Although the measured potential rates do not reflect *in-situ* activity of sulfate reducers, they show that microorganisms capable of employing sulfate reduction are still present in the deep coal-bearing sediments. This represents the deepest persistence of this type of microorganisms in marine sediments to date. The finding highlights the ability of sulfate reducers to survive over geological timespans even in sulfate-depleted environments, which might support the existence of a “paleome.” After having survived over more than 20 million years after burial, it might well be that sulfate reducers in the coalbeds to date have entered a dormant, spore-like state and were reactivated by the supply of excess sulfate during our incubations. Nevertheless, it is suggested from the strongly δ^34^S-enriched pyrite present in the coals, that they have been active long after burial and it might even be that a small fraction is still active operating at extremely low rates. The organic matter rich habitat provides significant amounts of energetically rich substrates (volatile fatty acids, methane, and hydrogen) and the availability of the electron acceptor sulfate is the obvious limitation for the survival of deeply buried sulfate reducers in the coalbed biosphere. A slow recycling of sulfate by Fe(III) might provide trace amounts of sulfate via re-oxidation and disproportionation and may have prevented a small population from dying out.

## Author contributions

CG designed the study; KH and FI led the expedition; FI, KH, CG, and NR collected the samples; CG measured pSRR in Exp.337 samples; FI measured pSRR in CK06-06 samples; NR and WG measured sulfur concentrations and isotopes; RA and KH measured hydrogen oxidation rates; CG wrote the paper; all co-authors reviewed the paper.

### Conflict of interest statement

The authors declare that the research was conducted in the absence of any commercial or financial relationships that could be construed as a potential conflict of interest.
