# Supplementary material for: Microbial Sulfate Reduction Potential in Coal-Bearing Sediments Down to ~2.5 km below the Seafloor off Shimokita Peninsula, Japan
Source: Front Microbiol. 2016 Oct 5;7:1576. doi: 10.3389/fmicb.2016.01576 (PMC5051215; doi:10.3389/fmicb.2016.01576)
Supplement: Supplementary file 1 [file DataSheet1.DOCX]

Supplementary Material

Potential microbial sulfate reduction up to 2.5 km below the seafloor in the deep coalbed biosphere off Shimokita (Japan)

**Clemens Glombitza*, Rishi R. Adhikari, Natascha Riedinger, William P. Gilhooly III, Kai-Uwe Hinrichs, and Fumio Inagaki**

*** Correspondence:** Corresponding Author: clemens.glombitza@bios.au.dk

# Supplementary Figures and Tables

## Supplementary Tables

**Supplementary Table 1.** Hydrogen oxidation rate measured by the tritium based hydrogenase enzyme essay (Adhikari et al., 2016), including core and section numbers, sample depth, sample lithology, lithological unit and maximum and minimum hydrogen oxidation rates of the sample replicates. Grey rows indicate samples from whole round core that were excluded from sulfate reduction rates due to possible contaminations.

| **Core-Section** | **Depth** | **Sample Lithology** | **Unit** | **Max. H_2_ oxidation rate** | **Min. H_2_ oxidation rate** |
| --- | --- | --- | --- | --- | --- |
|  | [mbsf] |  |  | [nmol H_2_ g^-1^ d^-1^] | [nmol H_2_ g^-1^ d^-1^] |
| 1R-1 | 1276.75 | Fine Sand | II | 1395 | 600 |
| 2R-2 | 1287.87 | Sandstone | II | 508 | 0 |
| 3R-2 | 1371.94 | Siltstone | II | 377 | 321 |
| 6R-1 | 1495.05 | Sandstone | II | 1809 | 0 |
| 8L-5 | 1607.26 | Shale | II | 160 | 0 |
| 9R-1 | 1625.56 | Sandstone | II | 382 | 129 |
| 10R-1 | 1630.16 | Siltstone | II | 1391 | 524 |
| 11R-1 | 1738.80 | Sandstone | II | 13 | 13 |
| 13R-4 | 1760.49 | Siltstone | II | 1070 | 357 |
| 14R-2 | 1822.41 | Siltstone | II | 1809 | 1743 |
| 15R-3 | 1921.98 | Lignite | III | 164 | 124 |
| 15R-6 | 1924.13 | Shale | III | 3579 | 2251 |
| 16R-3 | 1930.42 | Fine Sand | III | 0 | 0 |
| 18R-1 | 1945.71 | Lignite | III | 2520 | 721 |
| 19R-1 | 1950.04 | Sandstone | III | 4538 | 853 |
| 20R-5 | 1965.11 | Shale | III | 1226 | 1225 |
| 23R-3 | 1984.25 | Siltstone | III | 561 | 561 |
| 25R-2 | 1997.54 | Lignite | III | 563 | 563 |
| 25R-3 | 1998.75 | Silty Clay | III | 0 | 0 |
| 26R-4 | 2113.51 | Shale | IV | 1806 | 1771 |
| 27R-1 | 2200.91 | Shale | IV | 2796 | 1433 |
| 28R-4 | 2304.83 | Siltstone | IV | 233 | 0 |
| 28R-5 | 2305.34 | Siltstone | IV | 0 | 0 |
| 29R-5 | 2405.52 | Siltstone | IV | 715 | 473 |
| 30R-2 | 2447.61 | Lignite | IV | 761 | 0 |
| 30R-3 | 2449.43 | Shale | IV | 5201 | 2738 |
| 32R-1 | 2456.72 | Shale | IV | 1340 | 880 |

**Supplementary Table 2.** Sediment sulfur fractions (AVS: acid-volatile sulfide, CRS: chromium-reducible sulfur) and isotopic composition (δ^34^S in ‰ of Vienna Canyon Diabolo Troilite (VCDT) standard) of the CRS fraction containing the pyrites, core numbers, section numbers and lithological

unit.

| **Core-**  **Section** | **Depth** | **Unit** | **AVS** | **CRS** | **δ^34^S-CRS** |
| --- | --- | --- | --- | --- | --- |
|  | [mbsf] |  | [ppm] | [ppm] | [‰] |
| 2R-3 | 1289.52 | II | 0.07 | 880 | -3.2 |
| 3R-3 | 1372.675 | II | 0.56 | 6180 | -33.1 |
| 4R-3 | 1378.15 | II | 0.02 | 985 | 28.8 |
| 5R-3 | 1492.165 | II | 0.03 | 7711 | -3.9 |
| 6R-3 | 1496.48 | II | 0.08 | 6427 | -15 |
| 7R-1 | 1599.59 | II | 0.07 | 10753 | 3.9 |
| 8L-5 | 1607.435 | II | 0.52 | 72 |  |
| 8L-9 | 1611.25 | II | 0.03 | 6342 | -30.7 |
| 9R-4 | 1628.446 | II | 2.25 | 5422 | -29.4 |
| 10R-2 | 1631.525 | II | 0.02 | 6680 | -17.6 |
| 11R-3 | 1739.72 | II | 0.00 | 4880 | -37.2 |
| 11R-5 | 1743.165 | II | 0.02 | 1365 | -35.8 |
| 12R-6 | 1753.845 | II | 0.00 | 1545 | 0.8 |
| 12R-7 | 1754.76 | II | 0.00 | 5806 | -3.1 |
| 13R-2 | 1758.3525 | II | 0.00 | 7721 | 13.7 |
| 13R-8 | 1765.204 | II | 0.40 | 8271 | -4.5 |
| 14R-2 | 1822.535 | II | 0.02 | 9638 | -32.4 |
| 15R-2 | 1920.48 | III | 0.06 | 84 |  |
| 15R-5 | 1923.74 | III | 0.00 | 723 | 45.6 |
| 15R-7 | 1925.835 | III | 0.01 | 2681 | 29.3 |
| 16R-2 | 1929.6 | III | 1.35 | 2132 | 30.8 |
| 17R-1 | 1936.525 | III | 1.34 | 759 | 35.3 |
| 17R-4 | 1939.6 | III | 0.01 | 1049 | 27 |
| 17R-9 | 1944.315 | III | 0.00 | 1213 | 26.7 |
| 18R-1 | 1946.6 | III | 0.04 | 1952 | -13.4 |
| 19R-1 | 1951.181 | III | 0.00 | 1250 | 26.4 |
| 19R-5 | 1954.2585 | III | 1.68 | 1397 | 18.7 |
| 19R-8 | 1958.476 | III | 0.01 | 1843 | 33.2 |
| 20R-3 | 1961.63 | III | 0.00 | 3348 | 20.1 |
| 20R-7 | 1966.38 | III | 0.04 | 2093 | 43.3 |
| 21R-4 | 1971.775 | III | 0.38 | 2287 | 40.5 |
| 23R-8 | 1989.344 | III | 0.00 | 39.7 |  |
| 24R-3 | 1993.815 | III | 0.02 | 3.7 |  |
| 25R-1 | 1995.83 | III | 0.00 | 21.5 |  |
| 25R-2 | 1997.185 | III | 0.00 | 8.2 |  |
| 25R-3 | 1999.125 | III | 0.00 | 235 | 2.6 |
| 25R-4 | 1999.305 | III | 0.00 | 198 | 4.2 |
| 26R-2 | 2111.4255 | IV | 0.03 | 3265 | -28.6 |
| 26R-6 | 2115.657 | IV | 0.09 | 1947 | -21 |
| 26R-7 | 2117.8785 | IV | 0.00 | 4854 | -21.7 |
| 27R-2 | 2201.7 | IV | 0.03 | 3656 | -27.1 |
| 28R-3 | 2302.937 | IV | 0.00 | 1111 | 11.7 |
| 28R-6 | 2306.773 | IV | 0.00 | 710 | 23.9 |
| 29R-2 | 2401.618 | IV | 0.02 | 377 | 5.4 |
| 29R-5 | 2405.6475 | IV | 0.01 | 809 | -4.6 |
| 30R-4 | 2449.845 | IV | 0.00 | 3471 | 2.6 |
| 32R-2 | 2458.3215 | IV | 0.00 | 420 | 6.7 |

# Supplementary Movie

File name: C0020A-30R-2.mov

Movie file available via Figshare
Link: https://figshare.com/s/6ac50e6f31d5ce0b45f3

Description: Video clip of the computer tomography (CT) scan of core section 30R-2 containing the deep lignite layer showing the locations of fractures as well as the occurrence of pyrite (bright spots) in veins and granules.
